# The Immunogenetics of Psoriasis and Implications for Drug Repositioning

**DOI:** 10.3390/ijms18122650

**Published:** 2017-12-08

**Authors:** Xuan Xu, Hong-Yu Zhang

**Affiliations:** Hubei Key Laboratory of Agricultural Bioinformatics, College of Informatics, Huazhong Agricultural University, Wuhan 430070, China; xxpinky@webmail.hzau.edu.cn

**Keywords:** psoriasis, immunogenetics, genetics, susceptible genes, drug repositioning

## Abstract

Psoriasis is a genetically-regulated, T lymphocyte-mediated autoimmune skin disease that causes systemic damage, seriously affecting patient quality of life and survival. Psoriasis treatments, which aim to control the disease’s development, are greatly limited because its etiology and pathogenesis have not yet been fully elucidated. A large number of studies have demonstrated that immunogenetic elements are the most important factors responsible for psoriasis susceptibility. This paper delineates the immunogenetic mechanisms of psoriasis and provides useful information with regards to performing drug repositioning for the treatment of psoriasis.

## 1. Introduction

Psoriasis is a clinically common chronic inflammatory disease. Psoriasis patients experience skin tissue damage and can also simultaneously develop other systemic complications. This disease usually manifests with clinical and histological features, such as oval-shaped plaques with adherent, uplifted silver scales, dividing lines, and erythema. Scales are formed due to the incomplete keratinization of keratinocytes, which consists of prematurely overproliferated epidermal and cuticle-retained nuclei (parakeratosis). Compared to normal skin, an increased rate of mitosis in basal keratinocytes is observed. Since psoriasis is a skin-specific T-cell-mediated autoimmune disease, epidermal growth factors, nerve growth factors, adhesion factors, chemokines, neuropeptides, and T-cell receptors are involved in its pathogenesis. This disease affects approximately 1–3% of the world’s population [1]. Even after continuous developments and progress, the incidence of skin diseases continues to increase [2].

Epidemiological studies have established a genetic basis for psoriasis, with an estimated heritability of 60–90%, which is the highest among complex genetic diseases [3]. Familial studies have indicated higher incidences among primary and secondary relatives of psoriasis patients than among the general population, and three times the likelihood of incidence among identical twins than that of fraternal twins [4]. Approximately 70% of children with psoriasis present with family history of the disease [5]. Even though the genetic mechanisms of psoriasis are complex, a plausible explanation for its pathogenesis can be attributed to the abnormal activation of T cells and their migration into the skin, leading to the accumulation of inflammatory cells. CD4+ and CD8+ T cells then co-regulate the development of psoriatic lesions [5,6]. This hypothesis further confirms the importance of immunization in counteracting the pathogenicity of psoriasis.

Although the exact cause of psoriasis remains unknown, immune factors play a very important role in its pathogenesis. The use of immunotherapy has received increased attention due to the rapid progress in understanding the immune system-related psoriatic mechanisms. In fact, the psoriasis drug market is currently dominated by various types of antibody-based drugs [7]. Since psoriasis is a chronically recurrent disease, many patients require long-term treatment. However, up to 30% of the patients treated with anti-tumor necrosis factor (*TNF*) drugs (such as adalimumab) respond inadequately, and up to 50% lose response over time [8]. Even though anti-*IL-17A* drugs show better safety profiles compared to anti-TNF agents, they are often associated with an increased risk of severe infection and allergic, immunological, or other unwanted reactions [9]. Therefore, the biggest concern regarding these immunotherapies is with respect to their safety. The continued development of effective therapies for psoriasis is urgently needed. Drug repositioning (the application of approved drugs for new therapies) has provided an efficient route for drug discovery. It has the potential to greatly benefit affected patients by analyzing the immunogenetic mechanism of psoriasis. In this paper, following the analysis of the immunogenetic mechanism of psoriasis, we show the great potential of genetics-directed drug repositioning [10] in psoriasis treatment.

## 2. The Immunogenetics of Psoriasis

Like many immune-mediated diseases, psoriasis is associated with the major histocompatibility complex (MHC) alleles [11]. The ability of a series of immunosuppressive agents, such as cyclosporine, denileukin diftitox, and alefacept, to improve psoriasis has revealed the importance of the immune system in its pathogenesis. Psoriasis was once thought to be a disease associated with the *TNF*-mediated Th1-type immune response. Recently, the cellular and molecular contributions to the overactive immune response were further elucidated. The expression profiles of genes encoding Th1, Th2, and Th17 cytokines were extensively evaluated in differentiated skin cells of psoriatic patients. Th1 and Th22 cells were found to produce abundant psoriatic cytokines, such as *IL-17*, *IFN-γ*, *TNF*, and *IL-22*, which mediated the potentiation of keratinocytes on psoriatic inflammation. Although the role of Th1 cells in psoriasis is questioned, the use of cytokine antibodies in treatments has shown that *IL-23*, *TNF*, and *IL-17* play key roles in the pathogenesis of psoriasis [12,13].

More than 40 regions in the human genome have been associated with psoriasis [14], such as *HLA-Cw6*, *IL12B*, *IL23R*, *LCE3A*, *LCE3D*, and *STAT3C*. The chromosomal region that is thought to encode a psoriasis gene is referred to as the psoriasis susceptibility (PSORS) locus and is currently known to encode at least 15 different PSORS loci, which are mainly linked by a chain analysis of multiple members of the psoriasis family. The first gene found to be significantly susceptible to psoriasis was HLA-Cw6, which is located at PSORS1 with the chromosome position 6p21.3. Pathological studies have shown that HLA-C plays an important role in the antigen presentation of CD8+ T cells, and the CD8+ T cells in turn play a dominant role in the development and progression of psoriatic lesions. Therefore, HLA-C may be responsible for the expression profiles of genes important to psoriasis [15]. Studies have also shown that both the *ZNF816A* and *GJB2* loci are significantly associated with psoriasis in the German population, while *ERAP1* and *ZNF816A* are associated with type 1 (early-onset) psoriasis in the Chinese Han population. The disease susceptibility between the Chinese and European populations is heterogeneous, and may vary due to geographical differences, such as environmental factors affecting the disease susceptibility loci [16]. In general, after the classification of the associated genes, different types of biological mechanisms have been implicated in contributing to the etiology of psoriasis, including innate and adaptive immunity.

### 2.1. Genes Associated with Innate Immunity

The innate immune system provides an early response mechanism to host damage by identifying pre-formed non-specific effectors. There is evidence of psoriasis-associated inherent immune system disorders. For example, clinical observations indicate the importance of the congenital cytokine, interferon-alpha, as a psoriasis inducer [17].

NF-κB is a key regulatory element involved in a variety of intrinsic immunoregulatory and inflammatory pathways, cellular proliferation and differentiation, and apoptosis. Studies have confirmed that the NF-κB pathway is activated in psoriatic lesions and is downregulated after successful treatment [18]. We analyzed the genome-wide association study (GWAS) data and found that *TYK2*, caspase recruitment domain family member 14 (*CARD14*) [15], *NFK-BIA* [19], *TNFAIP3* [19], *TNIP1* [19], *IL-36RN* [20], and other genetic polymorphisms in this pathway are associated with the pathogenesis of psoriasis. *CARD14* expressed and regulated NF-κB in keratinocytes and was recently found to be a pathogenic gene of PSORS2 [21]. Moreover, all NF-κB proteins contain the Rel homologous domains that mediate DNA binding and dimerization.

Generalized pustular psoriasis (GPP) is often found in patients that exhibit or previously exhibited psoriasis vulgaris (PV). Interestingly, the interleukin-36 receptor antagonist gene *(IL-36RN*) is mainly expressed in the skin. It is largely responsible for encoding a soluble molecule, IL-36 receptor antagonist (*IL-36Rα*), which neutralizes the activation of NF-κB downstream signaling via the IL-36 receptors [20]. Animal experiments and population studies have found that most individual GPP cases were caused by homozygous or complex heterozygous mutations of *IL-36RN* [22,23].

RNF114 regulates innate immune system-related signaling molecules, such as *IL-1*, *IL-6*, and *IL-29*, which eventually results in the *EXOC2*-mediated production of *IF-1*. The *IF-1* gene encodes a viral RNA-activated apoptotic protein that initiates cellular antiviral and apoptotic responses upon sensing viral nucleic acids in the cytoplasm and protects the body by sensing and triggering the removal of virus-infected cells. The gene has been confirmed to be associated with psoriasis in both the Chinese and European populations [24].

Skin barrier function plays a dominant position in non-specific immunity, as the skin and mucous membranes constitute the body’s first immune barrier. Recently, researchers have focused on identifying new genetic markers associated with psoriatic skin barrier function. The terminal step of the keratinization process was associated with the formation of a highly specialized insoluble protein–lipid structure called the keratinized envelope (CE). The corneal capsule was essential for the normal functioning of the epidermal barrier. Thus, any abnormality in the expression of genes encoding portions of the envelope’s structure or of proteins involved in the enzymatic process may cause interference at different stages of keratinocyte differentiation, ultimately leading to the dysfunction of the epidermal barrier [25].

The epidermal differentiation complex (EDC) in the PSORS4 locus located on chromosome 1q21 contains genes that are expressed at various stages of the keratinization process. Currently, more than 45 genes have been identified in this complex. The EDC contains genes for epidermal regeneration (such as the S100 gene family) and terminally differentiated keratinocytes (small proline-enriched protein and advanced envelope protein (LCE protein)). These genes have different expression profiles in psoriasis. The LCE gene cluster is located in the PSORS4 locus of chromosome 1q21.3 and is a part of the EDC complex. More recently, the gene family encoding the late keratinized envelope (LCE) proteins has gathered increasing interest from researchers. The family can be divided into six subfamilies named LCE1–LCE6. Domestic and international scholars have confirmed that mutations or deletions in the LCE gene cluster (*LCE3B* and *LCE3C* gene single-nucleotide polymorphism (SNP) point) can cause skin barrier damage, leading to psoriasis [26].

### 2.2. Genes Associated with Adaptive Immunity

Adaptive immunity appears to be crucial for psoriatic pathogenesis, as the human leukocyte antigen (HLA) region is the most important susceptible site for psoriasis. Approximately 40% of the genes in the HLA region encode immune defense-related proteins [27]. A single-nucleotide polymorphism (SNP) possessing strong linkage disequilibrium (LD) along with the HLA-Cw6 allele produces the strongest correlation signal in all GWAS. Moreover, the HLA-Cw6 allele is also closely related to early-onset psoriasis in various races, which is supported by the fact that the antigenicity of the immune system is vital to the pathogenesis of psoriasis. Interestingly, a recent study has revealed a new pathway for the pathogenesis of psoriasis i.e., a melanocyte-specific response pathway where HLA-C*06:02 produces an autoimmune response against melanocytes by antigen presentation [28,29]. Additionally, the *ERAP1* gene is mainly involved in the processing of HLA class I molecules. Mutation of *ERAP1* affects the susceptibility of individuals carrying the HLA-C allele carriers, and studies have confirmed that the HLA-Cw6 allele is associated with this effect. A GWAS study showed a significant interaction between the HLA-Cw6 allele and rs27524-labeled *ERAP1* [30].

The role of *IL-23*/Th17 in psoriatic pathogenesis has become apparent in recent years with the discovery of the Th17 T cell subtype and the key Th17-polarized cytokine, *IL-23*. Many genes (e.g., *IL-12B*, *IL-23A*, *IL-23R*, *TRAF3IP2*, and *TYK2*) are significantly associated with psoriasis. Th17 cells are a type of novel effector T cell that secretes cytokines such as *IL-21* and *IL-17*. *IL-23* is a proinflammatory cytokine expressed in T cells, B cells, monocytes, mast cells, and endothelial cells. Both *IL-12B* and *IL-23R* have been validated in the Chinese population. A study found that *TNF* (SNP rs3093662) is a susceptible gene for psoriasis in the Chinese population [19]. Moreover, *IL-12* and *IL-23* contain a common p40 chain, and the *IL-12*p40 subunit binds to a unique subunit, p19, to form *IL-23* (p19/p40 dimer). The expression levels of the p19 and p40 genes in psoriatic lesions are increased, indicating that *IL-23* may play a major role in psoriatic lesions. Additionally, a GWAS study found that psoriasis was associated with mutations in *STAT3* (signal transduction and transcriptional activator 3), which is a key molecule in signaling cascades via several cytokines, including *IL-6*, *IL-10*, *IL-22*, and *IL-23*. Since *STAT3* is necessary for signal transduction via *IL-23R*, it is essential for Th17 polarization. Furthermore, psoriasis-related mutations in *STAT3* may reduce the threshold for the *IL-23* signal required to induce Th17 polarization. Targeted biologics have a high efficacy in psoriasis, and the high expression of *IL-23A* and *IL-12B* in psoriatic lesions also supports the above hypothesis regarding the pathogenesis of psoriasis [31,32].

Interestingly, associations of regions encoding Th2-related genes, particularly *IL-4*, *IL-5*, and *IL-13*, with the region of chromosome 5q31 containing multiple cytokine genes have also been found in patients with psoriasis [33]. *IL-4* and *IL-13* not only promote T cells toward Th2 differentiation, but also inhibit the development of mature Th17 cells. *IL-4* promotes both Th1 cell proliferation and *IL-23* expression, leading to a reduction in the number of Th17 cells. Although a number of studies have suggested that Th2 is not associated with psoriasis development, the strong correlation found between psoriasis and the GWAS between the genes encoding the cytokines *IL-4* and *IL-13* suggests a role for the Th2 pathway in psoriasis pathogenesis [33]. Erythrodermic psoriasis (EP) is among the most serious forms of psoriasis that affects human beings. The balance between Th1 and Th2 cells plays an important role in the pathogenesis of EP. Th1 cells induce the disease, while Th2 cells accelerate the inflammatory process, triggering the risk of infection. Th17/Treg may also lead to EP by binding to Th1 and Th2 cells (results summarized in Table 1) [34].

## 3. Implications for Drug Repositioning

Psoriasis treatments mainly aim to eliminate skin damage and prevent recurrence. Patients suffering from mild psoriasis are treated with local drugs and targeted light therapy. Patients with more severe forms of psoriasis undergo systemic treatments, such as a combination of methotrexate and phototherapy. However, its long-term use is hampered by safety issues. Understanding the progress of psoriatic immunogenetics is crucial in developing biotherapies that target the immune system, ideally for patients with intensive forms of the disease. The common biotherapies for the treatment of psoriasis include monoclonal antibody against interleukin *IL-12* and *IL-23* (ustekinumab) and anti-cytokine therapies (e.g., anti-tumor necrosis factor (*TNF*) therapies (adalimumab, etanercept, and infliximab)) [42].

Despite the progress in drug development, the approved drugs used to treat psoriasis have many limitations and defects. In spite of the strong anti-inflammatory effects of hormonal drugs (e.g., hydrocortisone acetate, prednisolone, betamethasone, and dexamethasone), external hormones are a temporary solution as long-term use may degrade the patients’ own hormone secretion glands, and lead to the re-emergence or a serious recurrence of the disease. Anti-tumor necrosis factor (TNF) drugs are first-line therapies for patients who exhibit immune tolerance under conventional systemic therapy. Although these treatments are relatively effective, about 30–50% of the patients respond inadequately [8,43]. Therefore, the development of new drugs to treat psoriasis has important research significance. A study has indicated that the medical genetics of drug targets may provide useful information with respect to drug repositioning, which involves predicting new activities and side effects of approved drugs [10]. This approach can accelerate the drug development process and reduce the associated risks. This paper mainly used the target-based approach to relocate a selection of approved drugs.

Analyzing the immune mechanisms and susceptibility genes of psoriasis not only deepens our understanding of the pathology, but also facilitates the use these susceptible genes as drug targets. The gain of function (GOF) and loss of function (LOF) mutations of pathogenic genes are the two major mechanisms that lead to the development of diseases. Therefore, drugs with appropriate modes of action (MoA) should be selected to treat the diseases i.e., inhibitors/antagonists should be used to combat GOF-induced diseases, whereas activators/agonists should be used to treat LOF-induced diseases [44].

We collected information on approved drugs that are used to treat psoriasis from the DrugBank (http://www.drugbank.ca), TTD (http://bidd.nus.edu.sg/BIDD-Databases/TTD/TTD.asp), and Clinical Trials (https://www.clinicaltrials.gov) websites. Furthermore, information regarding the GOF/LOF of pathogenic genes and the interactions between drugs and targets was collected from the OUGene website (http://www.csbio.sjtu.edu.cn). Using this method to reverse analyze, we found that many of these drugs met the requirements of the aforementioned pharmacological mechanisms (Table 2), thereby demonstrating the reliability of this strategy for fast and effective drug discovery.

Additionally, we also selected a number of important genes from the list of pathogenic genes mentioned above as drug repositioning targets by querying the literature.

Studies have shown that *STAT3* is the most common and easily activated STAT family member in human malignancies. *STAT3*-related skin diseases are characterized by epidermal hyperplasia and abnormal differentiation. *STAT3* performs its biological function by regulating several genes associated with apoptosis and proliferation and has been the focus of recent clinical research [45]. It is present in the cytoplasm of untreated cells and can be affected by the cytokines *IL-6* and *IL-10*, epidermal growth factor (*EGF*), hepatocyte growth factor (*HGF*), human epidermal growth factor receptor 2 (*HER2/Neu*), vascular endothelial growth factor (*VEGF*), and other ligands along with human erythropoietin (*EPO*) and human granulocyte-macrophage colony stimulating factor (*GM-CSF*), among other factors.

Psoriasis patients mainly express *VEGF* in the cytoplasms of keratinocytes from the basal layer to the granular layer. Contrastingly, *VEGF* has little or no expression in normal epidermis. In psoriasis patients, the *STAT3* protein is located in the cytoplasm and nuclei of the whole or middle layer of the epidermis, whereas *STAT3* is only present in the cytoplasm of the epidermal basal layer in normal epidermis. Therefore, *VEGF* and *STAT3* protein expression levels are higher in the skin lesions of psoriasis patients than in normal skin. Studies have shown that both proteins exhibit synergistic effects in psoriasis pathogenesis [46]. *EGFR* is a member of the epidermal growth factor family and is a transmembrane glycoprotein present on the surfaces of keratinocytes. It exhibits tyrosine kinase activity and plays important roles in the activation phases of proliferative signaling pathways. Its main ligands are epidermal growth factor (*EGF*) and *TGF-α*, among others; *TGF-α* can be formed by keratinocytes and then act in an autocrine manner, and *EGFR* can combine into dimers. Stimulation of the intrinsic tyrosine kinase function activates *EGFR*, which triggers downstream signal transduction pathways, such as RAS/ mitogen-activated protein kinases (MAPK), *P13/AKT*, and Janus kinase (JAK)/STAT, and it is found in many tumors. Drugs for *STAT3* are under investigation and have achieved satisfying therapeutic efficacy. One example is Benzo[b]thiophen-2-yl-3-bromo-5-hydroxy-5H-furan-2-one (BTH), a simple and interesting synthetic derivative of a natural compound separated from sponges, which can reduce the proliferation of keratinocytes by inhibiting the anti-inflammatory activity of the NF-κB signaling pathway and impair *STAT3* phosphorylation, preventing it from translocating to the nucleus and resulting in a decrease in keratinocyte proliferation [45].

As mentioned earlier, recent studies have also found interleukin 1β (*IL-1β*), tumor necrosis factor (*TNF*) [47], vitamin D receptor (*VDR*) [48], nitric oxide synthase 3 (*NOS3*) [49], and other genes to be overexpressed in psoriasis patients (GOF). Therefore, we used inhibitors for these targets and in turn designed drug repositioning strategies for psoriasis.

By performing psoriasis-related patent and literature searches, we found 12 of these drugs to be previously reported, further demonstrating our target-based drug repositioning method to have a high degree of accuracy and also indicating that the drugs we predicted by this method to be promising for psoriasis treatment (results summarized in Table 3).

## 4. Conclusions and Perspective

Psoriasis is an incurable disease that causes lifelong suffering in affected individuals. Currently, medical science faces challenges such as treatment failure, drug compliance, and drug side effects with respect to psoriasis treatments. In order to improve these deficiencies, we need a more effective treatment strategy. Rapid development in genomics and genetics has facilitated the identification of several psoriasis susceptibility genes and a comprehensive understanding of the immune mechanisms of psoriasis. Drug repositioning strategies based on immunogenetics can be used to effectively detect a drug’s potential in treating psoriasis and can even provide guidance for precision medicine. This approach can greatly influence the limitations of traditional treatments.

Given the current progress in science and technology, the analysis of single-nucleotide polymorphisms (SNPs) and copy number variants (CNVs) provides us with powerful tools to identify high-risk groups and disease-related genes, design and test medicines, and perform basic research in biology, which deepens our understanding of disease pathology and individual genetic differences. Moreover, the ability to study biological phenomena at omics levels is expected to lead to significant advances in precision medicine, as patients can be treated according to their own molecular characteristics. More importantly, the efficacy and toxicity of drugs can be predicted via genetic pharmacology and pharmacogenomics research, thus truly realizing the idea of personalized treatment. Therefore, by identifying differentially expressed genes (GOF or LOF) in each affected patient and using individual genome differences to guide medication, a specifically targeted drug can be chosen to achieve better therapeutic effects.

## Figures and Tables

**Table 1:** Genes associated with the immunogenetics of psoriasis.

Gene	Biologic Pathway	Protein Classification ^a^	Reference
B3GNT2	Adaptive immunity	Beta-1,3-*N*-acetylglucosaminyltransferase family	[15,19]
CARD14	Innate immunity; NF-κB signaling	Caspase recruitment domain-containing protein	[15,19,21,35]
CARM1	Innate immunity; NF-κB signaling	Protein arginine methyltransferase (PRMT) family	[19,21]
DDX58	Innate immunity; interferon gamma signaling	DEAD box proteins	[15,19]
EGFR	Adaptive immunity; organism-specific biosystem	Transmembrane glycoprotein	[36]
ELMO1	Innate immunity; signaling by PTK6	Member of the engulfment and cell motility protein family	[19]
ERAP1	Adaptive immunity; antigen presentation	Aminopeptidase involved in trimming HLA class I-binding precursors	[30]
ETS1	Adaptive immunity; immune response IL-23 signaling pathway	ETS family of transcription factors	[19]
EXOC2	Innate immunity; innate antiviral signaling	Component of the exocyst complex	[15,19,24]
FBXL19	Innate immunity; NF-κB signaling	Member of the Skp1-Cullin-F-box family of E3 ubiquitin ligases	[37]
HLA-C	Adaptive immunity; antigen presentation	HLA class I heavy chain paralogues	[15,19,30]
IFIH1	Innate immunity; innate antiviral signaling	DEAD box protein	[30]
IL-12B	Adaptive immunity; IL-23/Th17 axis	Subunit of interleukin 12	[30]
IL-13	Adaptive immunity; B-cell maturation and differentiation	immunoregulatory cytokine	[30,33]
IL-17A	Adaptive immunity; immune response IL-23 signaling pathway	Proinflammatory cytokine produced by activated T cells	[38]
IL23A	Adaptive immunity; IL-23/Th17 axis	Subunit of the heterodimeric cytokine interleukin 23 (IL23)	[15,30]
IL-23R	Adaptive immunity; IL-23/Th17 axis	Subunit of the receptor for IL23A/IL23	[30]
IL-28RA	Innate immunity; IFN signaling	Class II cytokine receptor family	[30]
IL-4	Adaptive immunity; Th2 signaling	Pleiotropic cytokine produced by activated T cells	[33,39]
IL-5	Adaptive immunity; Th2 signaling	Cytokine that acts as a growth and differentiation factor for both B cells and eosinophils	[33]
IL-36RN	Innate immunity; NF-κB signaling	Member of the interleukin 1 cytokine family	[22,23]
KLF4	Innate immunity	Kruppel family of transcription factors	[19]
LCE3B/LCE3C	Innate immunity; skin barrier function	Late Cornified Envelope (LCE)	[26,30]
MBD2	Adaptive immunity	Transcriptional repressor that binds to methylated DNA	[19]
NFKBIA	Innate immunity; NF-κB signaling	Member of the NF-κB inhibitor family	[19,30]
NOS2	Innate immunity	Reactive free radical	[15,19]
NOS3	Innate immunity; immune response Fc epsilon RI pathway	Reactive free radical	[40]
REL	Innate immunity; NF-κB signaling	Rel homology domain/immunoglobulin-like fold, plexin, transcription factor (RHD/IPT) family	[30]
RNF114	Innate immunity; innate antiviral signaling	Ring finger protein 114	[19]
RUNX3	Adaptive immunity; T-cell activation	Runt domain-containing family of transcription factors	[19]
SOCS1	Adaptive immunity; immune response IFN alpha/beta signaling pathway	Member of the STAT-induced STAT inhibitor (SSI)	[19]
STAT3	Adaptive immunity	STAT family of transcriptional activators	[15,19]
STAT5A	Adaptive immunity	STAT family of transcriptional activators	[15,19]
STAT5B	Adaptive immunity	STAT family of transcriptional activators	[15,19]
TAGAP	Adaptive immunity	Member of the Rho GTPase-activator protein superfamily	[19]
TNFAIP3	Innate immunity; NF-κB signaling	Zinc finger protein and ubiqitin-editing enzyme	[19]
TNFRSF9	Adaptive immunity	TNF-receptor superfamily	[19]
TNIP1	Innate immunity; NF-κB signaling	TNFAIP3 Interacting Protein 1	[30]
TRAF3IP2	Innate immunity; NF-κB signaling	Regulate responses to cytokines by members of the Rel/NF-κB transcription factor family	[30]
TYK2	Innate immunity; IFN signaling	Janus kinases (JAKs) protein families	[30]
UBE2L3	Innate immunity; NF-κB signaling	E2 ubiquitin-conjugating enzyme family	[19]
VDR	Innate Immunity; Vitamin D Metabolism, organism-specific biosystem	Nuclear hormone receptor for vitamin D3	[40]
VEGF	Innate immunity; immune response Fc epsilon RI pathway	Member of the PDGF/VEGF growth factor family	[41]
ZCH12C	Innate immunity	Zinc finger protein that regulates	[19]

^a^ Derived from NCBI Gene (https://www.ncbi.nlm.nih.gov/gene/). *TNF*: tumor necrosis factor; *STAT*: signal transduction and transcriptional activator; *VEGF*: vascular endothelial growth factor.

**Table 2:** Anti-psoriasis drugs and their medical genetics of targets.

Target	Genetic Disease Pathogenesis ^a^	Drug (Mode of Action) ^b^	Current Drug Indication ^c^
IL17-A	GOF	Secukinumab (inhibitor)	Ankylosing Spondylitis (AS); Psoriatic Arthritis; Severe Plaque psoriasis; Moderate Plaque psoriasis
IL17-A	GOF	Ixekizumab (inhibitor)	Severe Plaque psoriasis; Moderate Plaque psoriasis
NOS3	GOF	Prednisone (inhibitor)	Allergic Rhinitis (AR); Conjunctivitis, Seasonal Allergic; Psoriasis; Prostate Cancer; Lymphoma; Hodgkins Disease; Tuberculosis
NOS3/ARID5B	GOF	Methotrexate (inhibitor)	Severe Psoriasis; Systemic Lupus Erythematosus (SLE); Cancer, Breast; Acute Lymphocytic Leukemia (ALL)
STAT3	GOF	Acitretin (inhibitor)	Keratinization disorders; Severe Psoriasis
TNF	GOF	Infliximab (inhibitor)	Ankylosing Spondylitis (AS); Crohn's Disease (CD); Plaque Psoriasis; Psoriatic Arthritis; Rheumatoid Arthritis (RA); Ulcerative Colitis (UC)
TNF	GOF	Golimumab (inhibitor)	Ankylosing Spondylitis (AS); Rheumatoid Arthritis; Psoriatic Arthritis; Ulcerative Colitis
TNF/NOS3	GOF	Apremilast (inhibitor)	Psoriatic arthritis aggravated; Severe Plaque psoriasis; Moderate Plaque psoriasis
TNF/TNFRSF1B	GOF	Etanercept (inhibitor)	Ankylosing Spondylitis (AS); Graft Versus Host Disease (GVHD); Hidradenitis Suppurativa (HS); Plaque Psoriasis; Psoriatic Arthritis; Rheumatoid Arthritis
VDR	GOF	Atocalcitol (inhibitor)	Psoriasis
VDR	GOF	Tisocalcitate (inhibitor)	Psoriasis
VDR	GOF	Tacalcitol (inhibitor)	Psoriasis

^a,b^ Derived from OUgene (http://www.csbio.sjtu.edu.cn); ^c^ Derived from TTD, DrugBank, and ClinicalTrials. GOF: gain of function.

**Table 3:** Potential anti-psoriasis drugs predicted by medical genetics of targets and related literature/patents.

Target	Genetic Disease Pathogenesis ^a^	Drug (Mode of Action) ^b^	Current Drug Indication ^c^	Reference
EGFR	GOF	Zalutumumab (inhibitor)	Head and neck cancer	[50]
EGFR	GOF	Panitumumab (inhibitor)	Colorectal cancer	[51,52]
EGFR	GOF	Necitumumab (inhibitor)	Colorectal cancer	[53,54]
IL17-A	GOF	Vidofludimus (inhibitor)	Multiple sclerosis	n.a. ^d^
IL17-A	GOF	Cetuximab (inhibitor)	Colorectal cancer; cancer	[55,56]
IL1-B	GOF	Diacerein (inhibitor)	Rheumatoid Arthritis; Type 2 Diabetes Mellitus	[57]
IL1-B	GOF	Glucosamine (inhibitor)	Osteoarthritis	[58]
IL6	GOF	Ibudilast (inhibitor)	Allergic conjunctivitis	n.a.
STAT3	GOF	Atiprimod (inhibitor)	Inflammatory bowel disease	n.a.
STAT3	GOF	RTA 402 (inhibitor)	Solid tumours	n.a.
TNF	GOF	Amrinone (inhibitor)	Congestive heart failure	n.a
TNF	GOF	Pomalidomide (inhibitor)	Systemic sclerosis	[59] Patent No.: CN201310039583
TNF	GOF	Certolizumab (inhibitor)	Rheumatold arthritis	[60,61,62]
TNF	GOF	Lenalidomide (inhibitor)	Anaemia	[63,64]
VEGF	GOF	Bevacizumab (inhibitor)	Glaucoma	[65,66]
VEGF	GOF	Minocycline (inhibitor)	Bacterial infection	[67,68]
VEGF	GOF	Vandetanib (inhibitor)	Solid tumours	[69]

^a,b^ Derived from OUgene (http://www.csbio.sjtu.edu.cn); ^c^ Derived from TTD, DrugBank; ^d^ not available.
